# Vascular AGE-ing by methylglyoxal: the past, the present and the future

**DOI:** 10.1007/s00125-015-3597-5

**Published:** 2015-05-12

**Authors:** Casper G. Schalkwijk

**Affiliations:** Department of Internal Medicine, Laboratory for Metabolism and Vascular Medicine, Maastricht University Medical Centre (MUMC), Peter Debeyelaan 25, PO Box 5800, 6202 AZ Maastricht, the Netherlands; Cardiovascular Research Institute Maastricht (CARIM), Maastricht, the Netherlands

**Keywords:** AGEs, Diabetes, Glycation, Imaging, Methylglyoxal, RAGE, Risk prediction, Vascular complications, Vascular disease

## Abstract

Over the years, new research has elucidated the importance of the very fast formation of AGEs by the highly reactive methylglyoxal (MGO). It has become clear that MGO triggers maladaptive responses in vascular tissue. To counteract the deleterious effects of MGO, organisms have an enzymatic glyoxalase defence system in which MGO is converted to d-lactate, with glyoxalase 1 (GLO1) as the key enzyme in this system. Significant progress has been made towards the understanding of the MGO–GLO1 pathway in the pathogenesis of vascular disease in diabetes. This commentary highlights some lines of current research and future perspectives. The work conducted so far is only the starting point—in the coming 50 years, the MGO–GLO1 pathway will be the subject of intensified research, with special focus on pathophysiological pathways, the use of this system for early screening and risk prediction, and the development of intervention strategies for preventing vascular complications in people with and without diabetes. This is one of a series of commentaries under the banner ‘50 years forward’, giving personal opinions on future perspectives in diabetes, to celebrate the 50th anniversary of *Diabetologia* (1965–2015).

The latest estimates from 2013 show that 382 million people worldwide are suffering from diabetes and this is expected to rise to 592 million by 2035 [1]. The burden of diabetes is determined mainly by micro- and macrovascular endpoints. The increased formation of AGEs has been proposed as one mechanism that could explain the much faster development of vascular complications in diabetic than in non-diabetic individuals.

Traditionally, the formation of AGEs is viewed as a post-translational modification of proteins and other macromolecules by reduced sugars that accumulate slowly on extracellular and long-lived proteins throughout life. This reaction was first described in 1912 by food chemist Louis Camille Maillard. Although Maillard suggested that these chemical processes would be important in biology and medicine, it was not until the early 1980s that the pathophysiological significance of AGEs emerged in medical science [2]. Although the formation of AGEs is a naturally occurring process resulting from normal metabolism, it increases under hyperglycaemic conditions as well as under conditions of increased oxidative stress and hyperlipidaemia. AGEs are not inert; several mechanisms have been proposed to explain how AGEs contribute to the development of pathological conditions, including the aberrant crosslinking of extracellular matrix proteins, which results in a decrease of elasticity in blood vessels and leads to arterial stiffness, which is a hallmark of vascular ageing, as well as impaired permeability and cellular motility. In addition, AGEs bind to the cell-surface receptor RAGE (receptor for AGEs). This binding does not accelerate clearance or degradation but rather begins a sustained period of cellular activation that is mediated by receptor-dependent signalling, leading to inflammation-provoking tissue injury (Fig. 1).

**Fig. 1:**
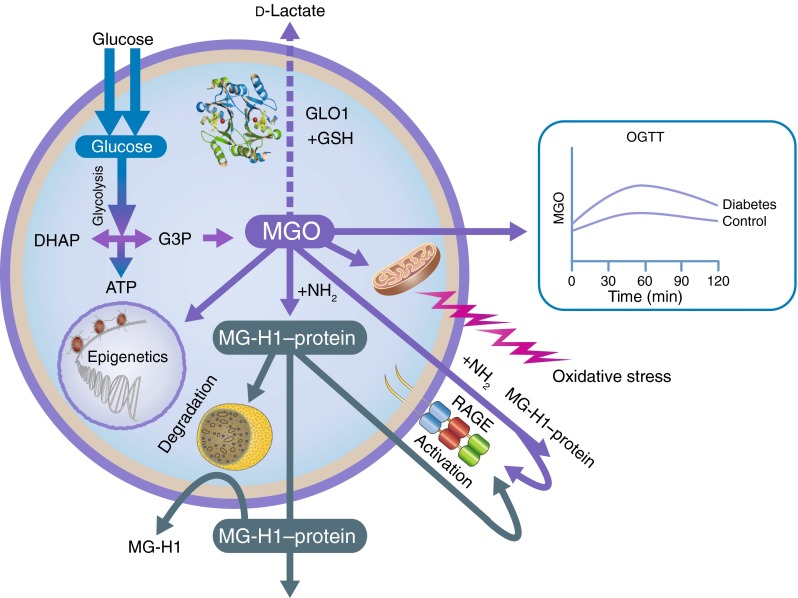
Biology of MGO: MGO is mainly formed as a byproduct of glycolysis. Increased levels of MGO are predominantly detoxified by GLO1, the key enzyme in the glyoxalase system, which converts MGO to d-lactate. Type 2 diabetes is associated with higher MGO incremental AUC, calculated from OGTT. MGO is involved in epigenetics and oxidative stress, and reacts, intracellularly and extracellularly, with arginine residues in proteins to produce MG-H1, the most important MGO-derived AGE. MG-H1 is cleared by lysosomal degradation. MGO modifications lead to protein inactivation, and changes in functionality and binding affinity to RAGE, resulting in maladaptive responses in vascular tissue. DHAP, dihydroxyacetone phosphate; G3P, glyceraldehyde 3-phosphate; GSH, glutathione

There have been dramatic transformations in AGE research in recent years. It was long believed that AGEs only accumulate very slowly on long-lived extracellular proteins, but new research has elucidated the significance of very fast AGE formation on cellular and short-lived extracellular proteins, lipids and DNA, with the highly reactive methylglyoxal (MGO) as a key compound involved in the very fast generation of glycation adducts [3]. MGO has been linked to a range of detrimental effects on cellular function, including increased oxidative stress. To counteract these effects, organisms have an enzymatic glyoxalase defence system that catalyses the conversion of MGO to d-lactate. Glyoxalase 1 (GLO1) is the key enzyme in this system and is a major line of defence against glycation. In recent years, remarkable progress has been made towards understanding the glyoxalase pathway, and MGO in particular, with the development of specific and sensitive methods for the assay of MGO [4], the demonstration that MGO is mainly generated as a byproduct of glycolysis and the demonstration that MGO is the most potent glycation agent [3]. The aim of this commentary is to further highlight the potential importance of MGO and GLO1 in risk prediction, pathogenesis and intervention in vascular disease in people with diabetes. Some lines of current research and future perspectives are debated.

## MGO and risk prediction of vascular disease

With increasing incidence of diabetes, the early detection of vascular disease continues to be a great challenge for clinicians in the 21st century. Despite the development of integrated risk scores for vascular disease, it is still very difficult to precisely estimate which individuals will develop vascular disease and which individuals will remain event-free. Even the extent to which high glucose levels in diabetic patients, as estimated by measuring HbA_1c_ levels, contributes directly to vascular disease remains controversial. Recent studies have suggested that postprandial spikes of high glucose may be a more robust determinant of vascular risk than average glucose levels as determined by HbA_1c_. However, we should recognise that HbA_1c_ levels are unlikely to provide a fully accurate picture of temporal glycaemic fluctuations, and thus to discriminate between diabetic individuals with and without regular occurrences of hyperglycaemic spikes. This may be one of the underlying reasons why glycaemic control, as measured by decreasing HbA_1c_ levels, has not been consistent in successfully reducing vascular disease.

Epidemiological studies support this concept, showing that glucose levels after an OGTT are an independent risk factor for cardiovascular disease (CVD), while fasting glucose levels are not, or are less so [5]. The formation of MGO might be a possible mechanism that causes repeated glucose spikes to have a more damaging effect than high fasting or mean glucose levels on endothelial cells and the development of vascular complications. We recently found that the presence of both impaired glucose metabolism and newly diagnosed type 2 diabetes were significantly associated with higher plasma MGO incremental AUC, as calculated by OGTT [6]. MGO levels would be expected to increase still further in individuals with known type 2 diabetes. Because experimental research has demonstrated that an excess of MGO initiates and augments retinopathy, nephropathy, neuropathy and atherosclerosis, it is conceivable that the effect of postprandial glucose spikes on vascular disease is due, at least partly, to MGO. If this is the case, MGO is a strong candidate for use as a more robust clinical marker of glycaemic fluctuation than HbA_1c_. MGO is derived from glucose but is up to 20,000 times more reactive with proteins. A major challenge for the future would be to systematically analyse MGO-modified proteins in order to identify one that is a better risk predictor than HbA_1c_.

Data from a recent cohort study have demonstrated that increased MGO-derived AGEs are associated with an increased risk for cardiovascular events in type 2 diabetic patients [7]. Furthermore, a large integrative genomic study identified GLO1 as a causal molecular mechanism in the development of CVD [8]. Whether we can improve risk prediction for vascular disease by including components of the MGO–GLO1 pathway in the risk engineering scores remains a key issue. A careful cataloguing of the proteomic alterations caused by MGO is likely to speed up the development of predictive biomarkers for vascular disease [9]. When we succeed in unravelling the MGO proteome, it will be an enormous boost for the early detection of vascular disease.

Another task for the coming decade is implementing new imaging tools to detect specific and well-characterised AGEs, as well as MGO, at the sites of complications. The technological progress in mass spectrometry imaging (MSI) provides these innovative possibilities. Using MSI to detect and quantify MGO, AGEs and GLO1 in human tissues within their histological contexts will provide unique insights into the role of AGEs in the pathogenesis and management of chronic diseases.

## MGO as a key factor in vascular complications

Although there is now considerable scientific evidence that MGO engenders the development of vascular complications, further research is needed to pinpoint the underlying causal mechanisms. MGO reacts predominantly with arginine residues on proteins, with methylglyoxal hydroimidazolone (MG-H1) being the most prevalent MGO-derived AGE modification found in vivo, leading to the structural change, inactivation and degradation of target proteins.

Several studies have shown a downregulation of GLO1 not only in the context of hyperglycaemia, but also in inflammatory signalling [10], hypoxia [10] and ageing [11], and this is accompanied by increased levels of MGO, indicating that this pathway is not only key for diabetic complications, but also for other age-related diseases such as atherosclerosis [10]]. The decline in GLO1 activity in arterial tissue may contribute directly to an increased risk of CVD with ageing. It is becoming clear that the glyoxalase and RAGE pathways are intertwined. MG-H1 has a very high (nanomolar) affinity for RAGE, while the binding of the best-characterised AGEs (i.e. *N*^ε^-(carboxymethyl)lysine-containing peptides) to RAGE is weak and in the micromolar range [12]. Various MG-H1 modified proteins as well free MG-H1 in tissue and plasma can bind to RAGE. The fact that the peptide backbone of the MG-H1-containing proteins is not involved in RAGE binding indicates that RAGE may act as a pattern recognition receptor. The RAGE-dependent downregulation of GLO1, which leads to increased MG-H1 formation on various proteins, will ultimately lead to further ligation and activation of RAGE, and to a continued and self-perpetuated inflammatory response. Targeting the formation of MGO and the RAGE interaction may therefore provide a novel therapeutic strategy in treating micro- and macrovascular complications and contribute to ‘successful vascular ageing’. This will be a topic to explore in the coming years.

To date, efforts to treat vascular complications have been hampered by ‘metabolic memory’, a phenomenon in which prior exposure to hyperglycaemia predisposes diabetic patients to the continued development of vascular disease, which persists even after improved glycaemic control. One possible explanation for this metabolic memory is that prolonged epigenetic changes in tissues lead to persistent changes in the expression of the genes associated with diabetic vascular complications. In addition to the modification of histones, changes in methylation patterns of the DNA itself have been described in the context of diabetes and epigenetic changes, and may also contribute to the development of metabolic memory. As stated, MGO has not only been linked to the modification of proteins, but has also been shown to directly modify and damage DNA. Indeed, a first link between the accumulation of MGO and the development of an epigenetic memory has been made [13]. Since epigenetic changes form the basis of many diseases, managing MGO levels could be the key to preventing multiple vascular complications.

## MGO as a target for intervention

From a clinical point of view, reducing the accumulation of MGO and enhancing GLO1 activity could provide new therapeutic opportunities for minimising the pathophysiological modifications associated with increased levels of MGO. Because MGO is mainly formed by the degradation of triose phosphate intermediates in glucose metabolism, improving metabolic control and reducing hyperglycaemia may be a first step in reducing the accumulation of MGO. Fortunately, new approaches to lowering glucose excursions have been developed, including the bionic pancreas, sodium–glucose co-transporter 2 (SGLT2) inhibitors and dipeptidyl peptidase-4 (DPP-4) inhibitors. Preliminary studies suggest benefits for cardiovascular risk, and it will be interesting to explore whether their mechanisms of action seem to be dependent on the reduction of MGO.

Several more pharmacological interventions have been developed to inhibit the accumulation of MGO, of which pyridoxamine is regarded as the most promising. Other potential treatments include arginine-containing peptides [14]. However, the strong affinity of MGO with arginine residues in vivo is a point of concern, and it is important to determine whether the effective affinity of pyridoxamine and arginine-containing peptides for MGO is strong enough to compete with endogenous arginine residues.

Another treatment strategy for reducing MGO is the use of GLO1 inducers. Since GLO1 expression is mediated via nuclear factor erythroid 2-related factor 2 (Nrf2) binding to the antioxidant responsive element (ARE) in the GLO1 promoter, activators of this Nrf2 transcription system are important for ongoing studies. Isothiocyanates, including sulforaphane, are interesting in this regard. These compounds, which can be found in cruciferous vegetables, are known to activate Nrf2. It has been demonstrated that Nrf2 activators increase the levels of GLO1 expression and decrease cellular and extracellular concentrations of MGO and MGO-derived protein adducts, while also reducing mutagenesis and cell detachment [15]. These findings highlight the importance of the regulatory increase in cell defences against MGO via the Nrf2–ARE–GLO1 pathway. Dietary bioactive inducers of GLO1 could therefore be used for patients with ageing-related disorders in which MGO plays a pivotal role.

A century ago, non-enzymatic glycation and the glyoxalase system were discovered. Over the years, it has become clear that MGO triggers maladaptive responses in vascular tissue. Over the coming 50 years, MGO, MGO-derived AGEs and the glyoxalase system will be subjects of intensified research, with particular focus on pathophysiological pathways, the use of this system for early screening and improving risk prediction, and the development of intervention strategies for preventing vascular complications in people with and without diabetes. The work carried out so far is only the starting point. Hopefully, 50 years from now we will know exactly how to decrease the burden of diabetes and its complications.

## Abbreviations

ARE: Antioxidant responsive element
CVD: Cardiovascular disease
GLO1: Glyoxalase 1
MG-H1: Methylglyoxal hydroimidazolone
MGO: Methylglyoxal
MSI: Mass spectrometry imaging
Nrf2: Nuclear factor-erythroid 2-related factor 2
RAGE: Receptor for AGEs

## References

[1] Guariguata L, Whiting DR, Hambleton I (2014). IDF Diabetes Atlas: global estimates of diabetes prevalence for 2013 and projections for 2035. Diabetes Res Clin Pract.

[2] Monnier VM, Cerami A (1981). Nonenzymatic browning in vivo: possible process for aging of long-lived proteins. Science.

[3] Shinohara M, Thornalley PJ, Giardino I (1998). Overexpression of glyoxalase-I in bovine endothelial cells inhibits intracellular advanced glycation endproduct formation and prevents hyperglycemia-induced increases in macromolecular endocytosis. J Clin Invest.

[4] Rabbani N, Thornalley PJ (2014) Measurement of methylglyoxal by stable isotopic dilution analysis LC-MS/MS with corroborative prediction in physiological samples. Nat Protoc 9:1969–1979 10.1038/nprot.2014.129 25058644

[5] Cavalot F, Pagliarino A, Valle M (2011). Postprandial blood glucose predicts cardiovascular events and all-cause mortality in type 2 diabetes in a 14-year follow-up: lessons from the San Luigi Gonzaga Diabetes Study. Diabetes Care.

[6] Maessen DE, Hanssen NM, Scheijen JL et al (2015) Post-glucose load plasma α-dicarbonyl concentrations are increased in individuals with impaired glucose metabolism and type 2 diabetes: The CODAM study. Diabetes Care 38:913–920 10.2337/dc14-2605 25710921

[7] Hanssen NM, Beulens JW, van Dieren S (2015). Plasma advanced glycation end products are associated with incident cardiovascular events in individuals with type 2 diabetes: a case-cohort study with a median follow-up of 10 years (EPIC-NL). Diabetes.

[8] Makinen VP, Civelek M, Meng Q (2014). Integrative genomics reveals novel molecular pathways and gene networks for coronary artery disease. PLoS Genet.

[9] Rabbani N, Thornalley PJ (2014). Dicarbonyl proteome and genome damage in metabolic and vascular disease. Biochem Soc Trans.

[10] Hanssen NM, Wouters K, Huijberts MS (2014). Higher levels of advanced glycation endproducts in human carotid atherosclerotic plaques are associated with a rupture-prone phenotype. Eur Heart J.

[11] Morcos M, Du X, Pfisterer F, Hutter H (2008). Glyoxalase-1 prevents mitochondrial protein modification and enhances lifespan in Caenorhabditis elegans. Aging Cell.

[12] Xue J, Ray R, Singer D (2014). The receptor for advanced glycation end products (RAGE) specifically recognizes methylglyoxal-derived AGEs. Biochemistry.

[13] El-Osta A, Brasacchio D, Yao D (2008). Transient high glucose causes persistent epigenetic changes and altered gene expression during subsequent normoglycemia. J Exp Med.

[14] Bierhaus A, Fleming T, Stoyanov S (2012). Methylglyoxal modification of Nav1.8 facilitates nociceptive neuron firing and causes hyperalgesia in diabetic neuropathy. Nat Med.

[15] Xue M, Rabbani N, Momiji H (2012). Transcriptional control of glyoxalase 1 by Nrf2 provides a stress-responsive defence against dicarbonyl glycation. Biochem J.

